# Effect of Intensive Blood Pressure Control on Cardiovascular Remodeling in Hypertensive Patients with Nephrosclerosis

**DOI:** 10.1155/2013/120167

**Published:** 2013-09-11

**Authors:** Otelio Randall, John Kwagyan, Tamrat Retta, Kenneth Jamerson, Velvie Pogue, Keith Norris, Muluemebet Ketete, Shichen Xu, Tom Greene, Xuelei Wang, Lawrence Agodoa

**Affiliations:** ^1^Howard University, College of Medicine, Washington, DC 20060, USA; ^2^Division of Cardiovascular Disease, Department of Medicine, Washington, DC 20060, USA; ^3^University of Michigan, Ann Arbor, MI 48109, USA; ^4^Harlem Hospital Center, New York, NY 10037, USA; ^5^Martin Luther King/Charles R. Drew University of Medicine, Los Angeles, CA 90059, USA; ^6^University of Utah, Salt Lake City, UT 84112, USA; ^7^The Cleveland Clinic Foundation, Cleveland, OH 44195, USA; ^8^National Institute of Health, Bethesda, MD 66420, USA

## Abstract

Pulse pressure (PP), a marker of arterial system properties, has been linked to cardiovascular (CV) complications. We examined (a) association between unit changes of PP and (i) composite CV outcomes and (ii) development of left-ventricular hypertrophy (LVH) and (b) effect of mean arterial pressure (MAP) control on rate of change in PP. We studied 1094 nondiabetics with nephrosclerosis in the African American Study of Kidney Disease and Hypertension. Subjects were randomly assigned to usual MAP goal (102–107 mmHg) or a lower MAP goal (≤92 mmHg) and randomized to beta-blocker, angiotensin converting enzyme inhibitor, or calcium channel blocker. After covariate adjustment, a higher PP was associated with increased risk of CV outcome (RR = 1.28, CI = 1.11–1.47, *P* < 0.01) and new LVH (RR = 1.26, CI = 1.04–1.54, *P* = 0.02). PP increased at a greater rate in the usual than in lower MAP groups (slope ± SE: 1.08 ± 0.15 versus 0.42 ± 0.15 mmHg/year, *P* = 0.002), but not by the antihypertensive treatment assignment. Observations indicate that control to a lower MAP slows the progression of PP, a correlate of cardiovascular remodeling and complications, and may be beneficial to CV health.

## 1. Introduction

Population and hospital-based studies [1, 2] have demonstrated that persistently elevated blood pressure (BP) levels increase the risk of cardiovascular (CV) events and subsequent CV mortality. Though the precise mechanisms of this association are still being investigated, several studies have detected a direct link between increased BP variability and target-organ damage [3–7]. The major target organs for the complications of elevated BP are the kidneys, heart, brain, and the arterial system. Study of the character of vascular alterations and the time course of such alterations is important to better understand the mechanism of hypertensive process. Retta and Randall [8] have suggested that target-organ damage is the result of an integrated effect of BP level as a function of time, whether it is continuously elevated or intermittent increases of pressure. Arterial stiffness is linked with changes in BP profile, characterized by isolated increase in systolic pressure and/or increase in pulse pressure (PP). Increased PP can result from an increase in systolic pressure and/or a decrease in diastolic pressure, which is typical for advanced arteriosclerosis and is responsible for the diastolic pressure stabilization or decline observed in aging [9].

Investigations in hypertension have generally focused on the steady components of blood pressure, such as mean arterial pressure (MAP), which is determined by cardiac output and peripheral vascular resistance [10, 11]. Pulse pressure, an indicator of the pulsatile component of the cardiac cycle, is a marker of the biophysical properties of the arterial system and cardiovascular remodeling. It has been demonstrated in observational studies that PP is directly associated with CV conditions including carotid atherosclerosis [12, 13], white matter lesions [14], coronary artery disease and congestive heart failure [15], and left ventricular hypertrophy (LVH) [16], and it has been implicated in the development and progression of large vessel atherosclerosis and small vessel disease [17, 18]. The PP magnitude has been related to CV complications, even when it oscillates, within the normal pressure range (BP < 140/90 mmHg).

The incidence of end-stage renal disease (ESRD) due to high BP has steadily increased over the years, and it disproportionately affects African Americans [19, 20]. Epidemiological studies have shown that damage of large conduit arteries is a major contributing factor to morbidity and mortality in patients with chronic kidney disease and in those with ESRD [21, 22]. There are limited prospective studies that have investigated the prognostic value of hypertension in patients with ESRD and very few were aimed at clarifying the impact of arterial pressure components on the incidence of cardiovascular complications in ESRD. The role of PP on CV risk in ESRD patients has been established in recent analyses of large dialysis databases [23, 24]. These studies found that at any given level of SBP, a higher PP was associated with increased risk of death in dialysis patients. 

The African American Study of Kidney Disease and Hypertension (AASK), a multicenter clinical trial, was designed to evaluate the impact of two levels of mean BP control (a calculated low mean arterial pressure, MAP ≤ 92 mmHg, and a calculated usual MAP = 102 to 107 mmHg) and three antihypertensive drug regimens on the progression of hypertensive kidney disease [25–28]. In this characterized cohort, over the entire study period, results showed no difference in renal outcomes in participants randomized to a lower MAP goal and a usual goal [25–28]. The data, however, provides an excellent opportunity for a secondary analysis to evaluate the impact of time-dependent changes in arterial properties and cardiovascular remodeling, specifically, spontaneous or unintentional changes in a nonmonitored BP component, PP, over a specified time period, during which minimum changes in therapy occurred. Recently, we found that there may be differential effects of intensive blood-pressure control on kidney disease progression in patients with and those without baseline proteinuria [29]. The purpose of this observational study thus was to examine (i) the association between unit changes of PP on composite CV outcomes, (ii) the association of unit changes of PP with new detection of LVH, (iii) the effect of intensive blood-pressure control on time-dependent changes of the arterial system and/or CV remodeling characterized by rate of change in PP, during a 24 month period in which minimum changes in therapy occurred.

## 2. Methods

### 2.1. Participants

The 1094 randomized participants in the AASK study were self-identified African Americans with hypertension between 18 and 70 years of age, with initial GFR between 20 and 65 mL/min per 1.73 m^2^ and with no other identified causes of renal insufficiency. Exclusion criteria included (i) diastolic BP (DBP) < 95 mmHg, (ii) known history of diabetes mellitus (fasting glucose ≥ 140 mg/dL or random glucose > 200 mg/dL), (iii) urinary protein to creatinine ratio greater than 2.5, (iv) accelerated and malignant hypertension within 6 months, (v) secondary hypertension, (vi) evidence of non-BP-related causes of renal disease, (vii) serious systemic disease, (viii) clinical congestive heart failure, or (ix) specific indication for or contraindication to a study drug or study procedure. The protocol and procedures were approved by the IRB of each center, and all participants read and gave written informed consent. Participants were enrolled between February 1995 and September 1998. Planned followup to the end of the study in September 2001 was 3 to 6.4 years.

### 2.2. Study Design

The AASK trial and study design have been described elsewhere [25–28]. Briefly, participants were randomized to one of two MAP goals (MAP defines as (1/3) × systolic BP + (2/3) × diastolic BP): a usual MAP goal defined as MAP of 102–107 mmHg (*n* = 554) or a lower MAP goal defined as MAP ≤ 92 mmHg (*n* = 540) [25–28]. Within each MAP group, subjects were further randomized to double blind treatment with one of three first-line antihypertensive drugs: a beta-blocker, metoprolol (*n* = 441), an ACE inhibitor, ramipril (*n* = 436) or a calcium channel blocker, amlodipine (*n* = 216). If the target BP could not be attained on the first line therapy, additional open label antihypertensive drugs were added sequentially (furosemide, doxazosin mesylate, clonidine, and then hydralazine hydrochloride or minoxidil). In this secondary analysis, patients requiring the addition of another medication or an increase in the current medication between months 9 and 35 were excluded in order to avoid a potential treatment effect on the rate of change in PP after they had stabilized at the onset of the analysis period.

### 2.3. Measurements and Laboratory Procedures

Blood pressure was assessed at protocol visits conducted at baseline, monthly during the first 6 months of followup, and every two months thereafter. Additional clinic visits with further blood pressure assessments were scheduled as needed to titrate the antihypertensive medications to bring the blood pressure level within the target range. At each blood pressure assessment three consecutive seated BP measurements were obtained using a Hawksley random zero sphygmomanometer after at least 5-minute rest, with the mean of the last 2 readings recorded. Pulse pressure was calculated as the difference between the average systolic and diastolic BP readings. 

A central laboratory measured serum and urinary levels of creatinine and protein as well as lipids. LVH was assessed at baseline and at 2-year intervals during followup by electrocardiography (EKG), using local readings based on the Cornell voltage criteria. 

### 2.4. Outcomes

A composite endpoint defined by the occurrence of cardiovascular death or the first cardiovascular hospitalization after randomization was designated as the main CV outcome for the trial. To determine the CV composite, events identified as potentially CV by the local clinical investigators, including hospitalizations and death from myocardial infarction, stroke, heart failure, revascularization procedures, and other cardiovascular events resulting in hospitalization, were reviewed, and a final classification was determined according to a prespecified protocol by a Cardiovascular Outcomes Committee that was blinded to randomization assignment. Because routine followup ceased once patients reached end stage renal disease (ESRD), only pre-ESRD CV events are included in the CV composite. 

In addition to the main CV composite outcome, disease progression of the heart is also evaluated in this paper by the development of EKG-based LVH at the 4-year assessment. The increase in PP from 9 months through the end of the patients' follow-up period is used as a surrogate of stiffening of the arterial system secondary to arteriosclerosis and/or CV remodeling.

## 3. Statistical Analysis

### 3.1. Cross-Sectional Analyses at Baseline

Univariate summaries of clinical and demographic characteristics included means and standard deviations or frequencies and percents, as appropriate. Multiple regression analysis was used to evaluate the cross-sectional relationship between PP and selected baseline variables, including age, gender, BMI, history of heart disease, lipid measures, and GFR.

### 3.2. Relationship of Selected Outcomes with Baseline and Follow-Up Blood Pressure

A series of exploratory analyses was performed to describe the association of selected CV and renal outcomes with baseline and follow-up BP indices. For these analyses, the mean follow-up systolic BP, diastolic BP, and PP were defined for each patient by the average of the BP parameter over all non-GFR BP assessments starting with the 4th month after randomization through the end of followup. In these analyses the BP indices were computed starting at the 4th month, rather than the 9th month, because in this case they are being evaluated as predictor variables rather than as response variables, so that it is advantageous to incorporate measurements early in follow-up. The analyses involving mean followup values of the BP indices were restricted to 1065 patients with at least one BP measurement after month 4. 

 Separate Cox regression analyses were performed to relate the main CV composite outcome to the baseline and mean follow-up values of the BP indices, with follow-up time censored at the occurrence of non-CV mortality, ESRD, or the end of the study. Finally, separate logistic regression analyses were performed to relate newly identified LVH at 4 years to the baseline and mean follow-up values of the three BP indices. The logistic regression analyses were conducted in a subgroup of 531 patients who remained in the study and provided EKG assessments at 4 years.

### 3.3. Effects of Intensive BP Control on PP

Longitudinal mixed effects analysis was performed to estimate the effect of the randomized BP goal and drug treatments assignments on the rate of change of PP (PP slope) measurements obtained at protocol visits from 9 months after randomization through the end of followup. The mixed effects model assumed a random intercept plus a 1st order autoregressive error structure to account for the correlation in pulse pressure measurements in the same patients over time. Robust “sandwich” standard error estimates were used. The slopes were assessed using data after 9 months because most of the modifications in drug regimens required to attain target blood pressures under the AASK design had occurred by this time. Additional analyses were done including MAP, SBP, and DBP in the model in order to determine the effects of these BP components on the rate of change in PP. All analyses are performed using SAS Version 9 (SAS Institute Inc., Cary, NC).

## 4. Results

### 4.1. Subject Characteristics

The 1094 randomized participants included 425 women (39%) and had a mean age of 55 years. Four hundred and twenty (38%) had LVH, 52% had a history of heart disease, and 29% were smokers. Additional baseline demographic and clinical characteristic of the study participants are displayed in Table 1. At baseline, there were no significant differences in these characteristics between the low BP group (MAP ≤ 92 mmHg) and the usual BP group (MAP of 102–107 mmHg). In the univariate analyses, baseline PP was associated directly with age, HDL, failure to obtain a high school degree, doubling of urine proteinuria, and inversely with uric acid, GFR, and hematocrit (*P* < 0.01 for each relationship). GFR, hematocrit, and level of educational attainment were no longer significantly associated with PP in the multivariable analysis. PP was not significantly associated with either gender or BMI in either the univariate or multivariable analyses.

### 4.2. Association of PP with Composite CV Outcome

Table 2 presents results of separate regression models that relate the main CV composite outcome to baseline and mean follow-up BP indices after adjustment for baseline factors. In these analyses, the composite CV outcome was significantly associated with both baseline and mean follow-up SBP and PP. In particular, the risk of the CV composite endpoint was 12% greater (RR = 1.12, CI = 1.02–1.23, *P* = 0.02) for every 10 mmHg increase in baseline PP and 28% greater (RR = 1.28, CI = 1.11–1.47, *P* < 0.01) for every 10 mmHg increase in mean follow-up PP. Composite CV outcomes were not significantly associated with baseline MAP but with mean follow-up MAP. The CV composite outcome was not significantly associated with either baseline DBP or mean follow-up DBP after adjustment for baseline factors.

### 4.3. Association of Pulse Pressure with New Incidence of LVH

Results of logistic regression models showed that a 10 mmHg higher mean followup PP was associated with a 26% increase in risk of a new indication of LVH by EKG between the baseline and 4-year assessments (RR = 1.26, CI = 1.04–1.54, *P* = 0.02) (Table 3). There was a similar association between newly identified LVH and mean follow-up SBP (RR = 1.26, CI = 1.06–1.51, *P* = 0.01) but neither with DBP nor MAP. Baseline BP indices were not associated with newly identified LVH at 4 years. These analyses of LVH were restricted to the subgroup of 531 patients who remained in the trial and provided an EKG reading at the 4-year assessment. 

## 5. Effect of Intensive BP Goal on PP

**Figure 1:**
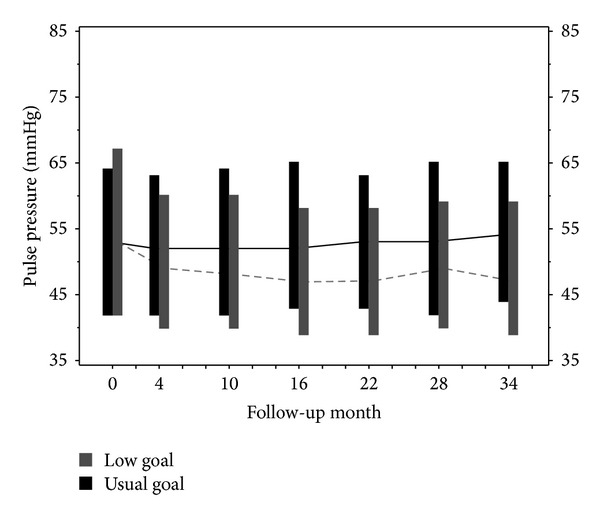
Trend in pulse pressure over time for the low and usual blood pressure groups. The median (connected by horizontal line), 25th percentile (low end of vertical bars) and 75th percentile (upper end of vertical bars) of the pulse pressure measurements at selected follow-up assessments are indicated. All available data were included at each time point. Numbers at the 10-, 22-, and 34-month assessments were 499, 447, and 412 for the low goal and were 497, 433, and 393 for the usual goal.

Figure 1 displays the distribution of PP over successive follow-up assessments for the two BP groups, with all available patients included at each time point.  Table 4 presents the results of the mixed effects analysis of the effects of the randomized treatment assignments on PP, SBP, DBP and MAP slopes starting at 9-month followup.

While the mean PP increased after 9 months in both MAP groups, after averaging across the three drug groups, the rate of increase (mean slope (*β*) ± SE) was significantly higher in the usual MAP goal compared to the lower MAP goal group (1.08 ± 0.15 versus 0.42 ± 0.15 mmHg per year, *P* = 0.002). Averaging across the two MAP groups, the mean rates of increase of PP in the 3 antihypertensive treatment groups were, respectively, 0.85 ± 0.15, 0.68 ± 0.17, and 0.71 ± 0.27 mmHg per year for ACE, BB, and CCB and did not differ significantly by drug treatment assignment, that is, no interaction between MAP goal and drug treatment assignment. The mean SBP declined significantly in the lower MAP group (overall mean slope −0.68 ± 0.20 mmHg per year), while there was no significant change in the usual MAP group (mean slope +0.21 ± 0.19 mmHg per year). The mean rates of change of DBP were negative and slightly steeper in the low than in the usual MAP groups, though not significantly different (−1.07 ± 0.13 versus −0.84 ± 0.11 mmHg per year, *P* = 0.16). This observation is important, because it shows that the effort of the study protocol to influence the MAP did not influence the BP components and subsequently PP.

The mean rates of change of mean MAP were also negative and significantly steeper in the low than in the usual MAP groups, (−0.95 ± 0.14 versus −0.49 ± 0.12 mmHg per year, *P* = 0.01). The larger increase in PP in the usual compared to the lower BP groups may have resulted from the larger difference between BP groups in the mean SBP slope than in the mean DBP slope.

## 6. Discussion

This observational study examines the impact of two achieved levels of MAP on the arterial system and cardiovascular remodeling, characterized by the rate of change in PP and the development of new LVH. In addition, we examined the effect of the two achieved MAP goals on the progression of PP. In our study elevated PP was associated with increased risk of CV events and development of LVH. Other studies [30–34] have reported similar findings. Mourad et al. [30] showed in patients with mild to moderate renal insufficiency that increased arterial stiffness of central arteries is associated with reduced creatinine clearance which was independent of blood pressure. Savage and colleagues [31] showed that in nondiabetic patients on maintenance hemodialysis, PP was a significant mediator of LVH. 

In the present study, progression of systolic BP was similarly associated with increased risk of CV events but minimally with development of LVH. Importantly, this study demonstrated that intensive control of BP significantly retarded the progression of PP. A recently reported data from AASK cohort [32] also indicates the long-term benefit of low MAP goal with ACE1 on progression of chronic kidney disease. We also found in a separate study that there may be differential effects of intensive blood-pressure control on kidney disease progression in patients with and those without baseline proteinuria [29]. These results, however, must be interpreted in the context of the primary and main secondary results of the AASK study [27, 28]. The trial was designed to achieve two different levels of mean arterial BP, the steady component of BP, and showed no significant benefit of the lower MAP goal compared to the usual MAP goal on either renal or CV outcomes. Input impedance, which completely characterizes the biophysical properties of the arterial system, includes steady hemodynamic measures such as MAP, cardiac output, and resistance as well as pulsatile components such as PP and stroke volume [33]. 

The mean arterial BP of the cardiac cycle has been considered an excellent choice to define the health status of the CV system [10, 11], especially since it defines the product of flow (i.e., cardiac output) and one index of arterial vascular property, total peripheral resistance. However, this expression largely ignores the pulsatile behavior of the flow from the heart and the status of the vascular properties of the proximal aorta, the initial site of LV-arterial system communication. Pulse pressure which is an “integrated” response to an impulse of flow, as well as largely a marker of the physiopathologic status of the proximal arterial system, is recognized as a predictor of CV outcomes [32, 33]. Nevertheless, whichever marker of systemic BP is used, the ultimate determinant of the pathological status and CV outcome should be the product of the magnitude and duration of the marker. This idea is supported by a large number of clinical and epidemiological studies indicating increasing mortality and morbidity with increasing stages and duration of hypertension [35, 36]. Several studies [37–40] in both man and animals have demonstrated that increased PP is associated with decreased arterial compliance. Such increase in PP amplitude is due to an increase in SBP and a decrease in DBP. Studies in chronically instrumented awake dogs [41, 42] have shown that BP increases for 4 weeks result in progressive weekly increase in PP from control to week 4. These data support the reliability and sensitivity of using the pulsatile component, PP as an indicator of changes in arterial system properties and functions in the two BP groups studied. 

The present study demonstrates the association between a marker of arterial vascular properties and cardiovascular remodeling, PP, and the development of LVH as well as a faster decline in renal function over time. In patients with ESRD, studies suggest arterial stiffness and early wave reflection to be the principal determinants of systolic and pulse pressures and are associated with LVH and its progression over time [34]. Increased arterial stiffness of the elastic type arteries is associated with reduced creatinine clearance in patients with chronic kidney disease [30, 43, 44]. In these patients, it has been suggested that the arterial system undergoes remodeling that is characterized by dilatation and, to a lesser degree, arterial intima-media hypertrophy of central, elastic-type, and capacitive arteries and isolated wall hypertrophy [43, 44]. Strengths of the present paper include a well-characterized cohort of patients who participated in a rigorously conducted, dose-targeted interventional trial. Moreover, this is one of a limited number of studies that examine an association of PP changes and CV outcomes in patients with hypertensive renal disease. 

### 6.1. Perspective

We believe the time-dependent vascular remodeling observations in this high-risk randomized population are important and deserve further investigation for the following reasons: (a) the PP, an integrated result of the pulsatile flow and arterial system interaction, is a real-time representation of the in vivo hemodynamics of hypertension and not just a mathematical mean as is the MAP; (b) the PP, an observed BP component, may be more sensitive for detecting vascular changes from hypertension than the randomized MAP, even at small BP differences; and (c) a better understanding of the shape and rate of increase in amplitude of the PP may improve the prevention and treatment of hypertension.

Two limitations must be acknowledged. First, because MAP was the independent variable in the trial, it is difficult to rule out the possibility that slower increase in PP after 9 months in the lower may have resulted from continued efforts of study personnel to bring MAP in the lower goal patients to ≤92 mmHg. We do not believe that was the case because there is always a close relationship among BP components except when the PP increases as a result of an increase in SBP and decrease in DBP. This possibility is suggested by the positive correlation of mean MAP with PP during the follow-up period (Pearson *R* = +0.25, *P* < 0.001), indicating that efforts to modify MAP may have also unintentionally modified PP to a limited extent and by the continued decline in SBP after 9 months in the lower but not in the usual MAP groups (see Table 4). However, this is unlikely since there was no significant decrease in the DBP after the 9th month and it would be difficult to selectively decrease SBP in an attempt to decrease MAP. Moreover, the protocol was not designed to influence the magnitude of PP, the oscillatory component of BP and a measure of the reflection of changes in arterial system properties as a function of time of the two different achieved MAP levels. Second, the exploratory analyses relating observed PP to CV outcomes are not based on randomized comparisons and are thus subject to the risk of bias due to uncontrolled confounding factors or reverse causality (i.e., effects of CV disease on PP). Nonetheless, while the present analysis does not establish a causative effect, the significant observation of association of PP with the CV outcomes is of clinical importance and deserves further research.

In conclusion, this study demonstrates an independent association of PP with cardiovascular outcomes and showed that PP progression over time was less when a lower BP goal was targeted compared to usual BP goal.

## Figures and Tables

**Table 1:** Baseline and clinical characteristics of study participants (*N* = 1094) mean SD or frequency (%).

Baseline characteristic*	Low BP goal	Usual BP goal
Age, years	54.5 ± 10.9	54.7 ± 10.4
Women	206 (38%)	219 (40%)
BMI (kg/m^2^)	30.5 ± 6.7	30.6 ± 6.5
BP (mmHg)		
SBP	152 ± 25	149 ± 23
DBP	96 ± 15	95 ± 14
MBP	115 ± 17	113 ± 15
GFR (mL/min per 1.73 m^2^)	46.8 ± 13.3	46.05 ± 13.9
Serum creatinine	1.98 ± 0.7	2.02 ± 0.7
Presence of LVH	225 (42%)	195 (35%)
History of heart disease	294 (54%)	270 (49%)

BMI: body mass index; BP: blood pressure; SBP: systolic blood pressure; DBP: diastolic blood pressure; GFR: glomerular filtration rate; LVH: left ventricular hypertrophy.

**Table 2:** Association of CV composite outcome with blood pressure indices.

BP index	Time period for blood pressure assessment	RR (95% CI)	*P* value
Diastolic BP (per 10 mmHg)	Baseline	1.07 (0.94–1.21)	0.30
	Mean follow-up	1.20 (0.97–1.49)	0.09
Systolic BP (per 10 mmHg)	Baseline	1.09 (1.01–1.17)	0.02
	Mean follow-up	1.30 (1.15–1.48)	<0.01
Pulse pressure (per 10 mmHg)	Baseline	1.12 (1.02–1.23)	0.02
	Mean follow-up	1.28 (1.11–1.47)	<0.01

Association of CV composite outcome with BP indices after adjustment for 11 baseline factors: age, gender, total cholesterol, HDL cholesterol,  uric acid, BMI, GFR, HCT (%), smoking status, education, and log transformed urine protein/creatinine ratio. Analyses of baseline BP indices were also adjusted for randomized treatment assignment. CV composite outcome was defined as a CV death or the first occurrence of a CV hospitalization. Sample sizes were 1080 (with 147 events for the CV composite) for analyses of baseline BP indices and 1052 (with 143 events for the CV composite) for analyses of mean follow-up values of BP indicates.

**Table 3:** Association of  new incidence of  LVH by year 4 with blood pressure  (baseline, *N* = 531 (110 events), mean follow-up, *N* = 530 (110 events)).

	Time period for blood pressure assessment	OR (95% CI)	*P* value
Diastolic BP (per 10 mmHg)	Baseline	1.15 (0.97–1.37)	0.112
	Mean follow-up	1.14 (0.85–1.55)	0.38
Systolic BP (per 10 mmHg)	Baseline	1.08 (0.98–1.20)	0.12
	Mean follow-up	1.26 (1.06–1.51)	0.01
Pulse pressure (per 10 mmHg)	Baseline	1.06 (0.92–1.22)	0.41
	Mean follow-up	1.26 (1.04–1.54)	0.02

Baseline covariates are: age, gender, total cholesterol, HDL cholesterol,  uric acid, BMI, GFR, HCT (%), smoking status, education, and urine protein/creatinine ratio.

**Table 4:** Mean (SE) rate of change of blood pressure measurements after 9 months of follow-up by randomized treatment assignment (mmHg/year).

Treatment assignment	Both BP groups	Low BP goal	Usual BP goal	*P* value for low versus usual BP
Pulse pressure				
ACE	0.85 (0.15)	0.57 (0.22)	1.13 (0.21)	
BB	0.68 (0.17)	0.38 (0.25)	0.99 (0.23)	
CCB	0.71 (0.27)	0.24 (0.39)	1.18 (0.38)	
All drug groups	0.75 (0.11)	0.42 (0.15)	1.08 (0.15)	0.002
Systolic BP				
ACE	−0.02 (0.22)	−0.38 (0.32)	0.34 (0.31)	
BB	−0.25 (0.21)	−0.68 (0.31)	0.18 (0.28)	
CCB	−0.64 (0.32)	−1.28 (0.47)	−0.01 (0.45)	
All drug groups	−0.24 (0.14)	−0.68 (0.20)	0.21 (0.19)	0.001
Diastolic BP				
ACE	−0.84 (0.14)	−0.90 (0.21)	−0.78 (0.18)	
BB	−0.88 (0.12)	−1.01 (0.17)	−0.76 (0.17)	
CCB	−1.33 (0.21)	−1.54 (0.32)	−1.12 (0.26)	
All drug groups	−0.96 (0.08)	−1.07 (0.13)	−0.84 (0.11)	0.16

*Analysis based on pulse pressures at non-GFR protocol visits from 9 months after randomization through the end of follow-up. The analysis was restricted to 1043 patients with at least 1 blood pressure measurement after 9 months and was performed without covariate adjustment.
